# Leukopenia and leukocytosis as strong predictors of COVID‐19 severity: A cross‐sectional study of the hematologic abnormalities and COVID‐19 severity in hospitalized patients

**DOI:** 10.1002/hsr2.1574

**Published:** 2023-09-27

**Authors:** Fateme Sharafi, Reza Jafarzadeh Esfehani, AmirAli Moodi Ghalibaf, Lida Jarahi, Ali Shamshirian, Mahnaz Mozdourian

**Affiliations:** ^1^ Department of Internal Medicine Mashhad University of Medical Science Mashhad Iran; ^2^ Blood Born Infections Research Center, Academic Center for Education Culture and Research (ACECR)—Khorasan Razavi Mashhad Iran; ^3^ Student Research Committee, Faculty of Medicine Birjand University of Medical Sciences Birjand Iran; ^4^ Department of Community Medicine, Faculty of Medicine Mashhad University of Medical Sciences Mashhad Iran; ^5^ Student Research Committee, Faculty of Medicine Mashhad University of Medical Sciences Mashhad Iran; ^6^ Lung Diseases Research Center Mashhad University of Medical Science Mashhad Iran

**Keywords:** COVID‐19, dyspnea, hematology test, Iran, leukocytosis, leukopenia

## Abstract

**Background and Aims:**

Predicting severe disease is important in provocative decision‐making for the management of patients with the coronavirus disease 2019 (COVID‐19); However, there are still some controversies about the COVID‐19's severity predicting factors. This study aimed to investigate the relationships between clinical and laboratory findings regarding COVID‐19's severity in patients admitted to a tertiary hospital in Mashhad, Iran.

**Methods:**

A cross‐sectional study was conducted on patients with documented COVID‐19 infection based on the reverse transcription‐polymerase chain reaction test. Clinical symptoms, vital signs, and medical history of the patients were recorded from their medical records. Laboratory findings and computed tomography (CT) study findings were documented. Disease severity was defined based on CT scan findings.

**Results:**

A total of 564 patients (58.8 ± 16.8 years old) were evaluated. The frequency of severe disease was 70.4%. There was a significant difference in heart rate (*p* = 0.0001), fever (*p* = 0.002), dyspnea (*p* = 0.0001), chest pain (*p* = 0.0001), diarrhea (*p* = 0.021), arthralgia (*p* = 0.0001), and chills (*p* = 0.044) as well as lymphopenia (*p* = 0.014), white blood cell count (*p* = 0.001), neutrophil count (*p* < 0.0001), lymphocyte count (*p* < 0.0001), and prothrombin time (*p* = 0.001) between disease severity groups. Predictors of severe COVID‐19 were pulse rate (crude odds ratio [cOR] = 1.014, 95% confidence interval [CI] for cOR: 1.001, 1.027) and leukopenia (cOR = 3.910, 95% CI for cOR: 1.294, 11.809). Predictors for critical COVID‐19 were pulse rate (cOR = 1.075, 95% CI for cOR: 1.046, 1.104), fever (cOR = 2.516, 95%CI for cOR: 1.020, 6.203), dyspnea (cOR = 4.190, 95% CI for cOR: 1.227, 14.306), and leukocytosis (cOR = 3.866, 95% CI for cOR: 1.815, 8.236).

**Conclusions:**

Leukopenia and leukocytosis have the strongest correlation with the COVID‐19 severity. These findings could be a valuable guild for clinicians in COVID‐19 patient management in the inpatient setting.

## 1 BACKGROUND

The coronavirus disease 2019 (COVID‐19) is caused by severe acute respiratory syndrome coronavirus 2 (SARS‐CoV‐2) which is similar to the previous coronavirus disease outbreaks including SARS and the Middle East Respiratory Syndrome (MERS). The COVID‐19 outbreak had considerable mortality and morbidity in almost every country.^1^, ^2^, ^3^ In contrast to the same period in 2018, the recent coronavirus disease was the third leading cause of death.^4^ The infection may be presented with a heterogeneous clinical presentation ranging from a mildly symptomatic disease to even a severe disease requiring intensive care unit (ICU) admission.^2^, ^5^ The common clinical symptoms are usually influenza‐like manifestations including fever, cough, and dyspnea while other nonrespiratory symptoms including cutaneous and gastrointestinal manifestations are prevalent.^6^, ^7^ Most of the severe symptoms leading to ICU admission and mortality are because of the excessive release of inflammatory markers and cytokines during the “cytokine storm” phase of the disease‐causing severe lung damage and respiratory failure.^8^, ^9^ Such severe clinical presentations provided logistic challenges for healthcare facilities, forcing them to increase their ICU capacities.^10^, ^11^ Therefore, the prediction of clinical outcomes and the determination of patients who are at risk of developing severe symptoms requiring ICU admission will result in more accurate scenario planning.^4^ Even more, the determination of disease severity and prognosis facilitates prioritizing vaccination plans for high‐risk populations.^4^, ^12^

For a significant duration, there were no curative treatment and prevention strategies to decrease the rate of infection^13^, ^14^, ^15^, ^16^; However, recent advances led to medications that significantly reduced COVID‐19 mortality. Additionally, vaccination is known as the most successful preventive strategy to date.^17^ Recent studies suggested some risk factors influencing the risk of severe infection and death from COVID‐19 and the determination of such risk factors helps to identify the high‐risk population and facilitates providing timely management. There are conflicting reports regarding the specific risk factors of severe COVID‐19 infection in different populations and a recent meta‐analysis study demonstrated that male gender, hypertension, smoking history, diabetes, and myalgia are predictors of COVID‐19 severity. However, factors including diarrhea, fever, and cough were not related to the disease severity.^18^, ^19^ Additionally, there are controversies about laboratory findings including hematological parameters as the predictors of COVID‐19 severity and their relationship with the disease outcomes.^20^

Regarding the lack of enough regional reports indicating the relation between different clinical and laboratory findings in COVID‐19 patients with different clinical severity, the present study aimed to investigate the relationships between clinical and laboratory findings with COVID‐19's severity in the patients admitted to a tertiary hospital in the northeast of Iran.

## 2 METHODS

### 2.1 Study design

During 10 months since the start of the pandemic in Iran (mid‐February 2020), every patient who was admitted with positive real‐time polymerase chain reaction (PCR) for SARS‐CoV‐2 infection enrolled in the present cross‐sectional study. The inclusion criteria were documented COVID‐19 infection based on RT‐PCR, being admitted in the tertiary hospital from May 2020 to August 2020. Patients with incomplete medical records were excluded from the study.

### 2.2 Outcomes

Clinical symptoms of every patient including fever, cough, weakness, headache, hemophilia, diarrhea, nausea and vomiting, dyspnea, and abdominal pain were recorded in a checklist. Also, patients’ medical history of any medical illness and medications were recorded. Vital signs including body temperature, blood pressure, breath rate, and pulse rate as well as oxygen saturation were evaluated for every patient. Bradycardia was defined as a heart rate below 60/min, tachycardia as a heart rate above 100/min, hypertension as blood pressure above 140/90 mmHg, and hypotension as blood pressure below 90/60 mmHg.

Laboratory studies including complete blood count, coagulation markers, D‐dimer, ferritin, erythrocyte sedimentation rate (ESR), and C‐reactive protein (CRP) alongside computed tomography (CT) study findings including the number of involved lobes were documented. Leukocytosis was defined as a white blood cell (WBC) count higher than 10,000/µL, leukopenia was defined as WBC less than 4000 µL, neutropenia was defined as a neutrophil count below 1500 µL, and lymphopenia was defined as lymphocyte count below 1500 µL.

The patient's disease severity was evaluated based on the CT scan criteria based on the findings of the study by Francone et al.^21^ Disease severity was defined using a scoring system based on the location and extent of pulmonary involvement. Scores below 7 were considered as mild, scores between 8 and 15 as moderate, and scores higher than 16 as severe.^21^

The clinical, laboratory, and imaging findings for every patient were compared among patients with different disease severity. Till the end of the study duration, three peaks in the prevalence of the disease were recorded. The first peak occurred from February to June 2020, the second peak occurred from June to September 2020, and the third peak occurred from September to December 2020.

### 2.3 Ethical issues

The present study has been approved by the Mashhad University of Medical Sciences Ethics Committee and took place in Imam Reza Hospital (Mashhad, Iran) (registration code: IR.MUMS.MEDICAL.REC.1399.485). Written informed consent was obtained from each patient.

### 2.4 Statistical analysis

Data analysis was performed using the statistical package for social sciences (SPSS) software version 16.0. The normality of the continuous variables was analyzed using the Kruskal–Wallis test. Due to the nonnormality of the data, median and interquartile range were used and the Kruskal–Wallis and Mann–Whitney tests were used to compare variables between groups. Categorical variables were presented using frequency and percentage and the chi‐square or Monte Carlo tests were used to compare these variables between groups. To identify the variables that could predict severity categories of COVID‐19, all variables that had p values below 0.20 in the comparison analyses were entered into the multinomial regression model. Disease severity was considered as the dependent variable with mild/moderate COVID‐19 severity considered as a reference and all other selected variables as independent variables to perform the regression model. The regression results were presented using crude odds ratio (cOR) and 95% confidence interval (CI) for OR. The level of statistical significance was set as *p* < 0.05.

## 3 RESULTS

A total of 564 admitted patients with COVID‐19 were included in this study. The majority of patients were admitted during the second peak (217, 38.8%) of the disease followed by the first and third peaks of 206 (36.8%) and 137 (24.5%) patients, respectively. The mean age of the study patients was 58.8 ± 16.8 years old and the majority of the patients (339, 60.1%) were male. Demographic characteristics and disease history of the study patients are presented in Table 1. Most of the patients were using angiotensin‐converting enzyme inhibitors/angiotensin receptor blockers (242 patients, 43%) and 222 (12.5%) were using antidiabetics, 15 (2.6%) using nonsteroidal antiinflammatory drugs, 12 (2.1%) patients were receiving chemotherapy or immunosuppressive agents and 6 (1%) patients were using prednisolone.

**Table 1:** Demographic characteristics and disease history of the patients in this study.

Variable	Mean ± *SD*	Frequency (%)
Age (years)	58.8 ± 16.8	–
Gender		
Male	–	339 (60.1)
Female	–	225 (39.9)
Smoking	–	145 (25.7)
Addiction	–	56 (9.9)
Alcohol use	–	3 (0.5)
Hypertension	–	295 (52.3)
Diabetes	–	227 (40.2)
Ischemic heart disease	–	162 (28.7)
Chronic obstructive pulmonary disease	–	67 (11.9)
Chronic kidney disease	–	40 (7.1)
Malignancy	–	26 (4.6)
Cardiovascular disease	–	15 (2.7)
Asthma	–	13 (2.3)
Organ transplantation	–	11 (2.4)
Congestive heart disease	–	9 (1.6)
Hypothyroidism	–	7 (1.2)
Morbid obesity	–	5 (0.8)
Pulmonary thromboembolism	–	4 (0.7)
Cholecystitis	–	4 (0.7)
Tuberculosis	–	3 (0.5)
Rheumatoid arthritis	–	2 (0.4)
Lupus	–	2 (0.4)
Schizophrenia	–	2 (0.4)
Multiple sclerosis	–	2 (0.4)
Goodpasture syndrome	–	1 (0.2)
Alport syndrome	–	1 (0.2)
Thrombotic thrombocytopenic purpura	–	1 (0.2)
Psoriasis	–	1 (0.2)
Necrotizing fasciitis	–	1 (0.2)
Fractures	–	1 (0.2)
Pyelonephritis	–	1 (0.2)
Appendicitis	–	1 (0.2)
Hepatitis B	–	1 (0.2)

Based on CT scan criteria, severe disease was identified in 397 (70.4%) and critical disease was identified in 144 (25.5%) patients. Clinical presentations and disease severity of the study subjects are presented in Table 2. The most common severity criteria in the patients in our study were low O_2_ saturation (524, 93.2%) followed by high respiratory rate (492, 88.2%). ICU admission was recorded for 146 (26.3%) patients. Based on clinical data 546 (96.8%) patients were categorized as severe COVID‐19, while both the laboratory data and CT‐scan findings determined severe COVID‐19 in 151 (27.0%) patients (Table 2).

**Table 2:** Clinical presentations and disease severity of the patients in the study.

Variable	Frequency	Percentage
O_2_ saturation <93%	524	93.2
Respiratory rate >24/min	492	88.2
ICU admission	146	26.3
Positive clinical criteria		
1 criterion	63	11.9
2 criteria	328	61.8
3 criteria	140	26.4
Disease severity based on clinical data		
Mild/moderate	18	3.2
Severe	546	96.8
CRP >50	392	69.5
Elevated erythrocyte sedimentation rate (ESR)	361	64.0
D‐Dimer >1000	51	9.0
Ferritin >1000	19	3.4
Disease severity based on laboratory data		
Mild/moderate	408	73.0
Sever	151	27.0
Computerized scan (CT scan) score		
Mild (<7)	108	19.3
Moderate (8–15)	300	53.7
Severe (>16)	151	27.0

Abbreviation: ICU, intensive care unit.

A comparison of clinical findings between disease severity groups in the study patients is presented in Table 3. There was a significant difference in heart rate (*p* = 0.0001), fever (*p* = 0.002), dyspnea (*p* = 0.0001), chest pain (*p* = 0.0001), diarrhea (*p* = 0.021), arthralgia (*p* = 0.0001), and chills (*p* = 0.044) between disease severity groups.

**Table 3:** Comparison of clinical findings in‐between disease severity groups in patients in the study.

Variable	Mild/moderate frequency (%)	Severe frequency (%)	Critical frequency (%)	*p* value
Pulse rate				
Bradycardia	0 (0.0)	4 (1.0)	1 (0.7)	0.0001^a^^,^ ^b^
Normal	17 (94.4)	137 (34.5)	24 (16.7)	
Tachycardia	1 (5.6)	256 (64.5)	119 (82.6)	
Systolic blood pressure				
Hypotension	0 (0.0)	5 (1.3)	0 (0.0)	0.192^a^
Normal	10 (58.8)	154 (38.9)	49 (34.5)	
Hypertension	7 (41.2)	237 (59.8)	93 (65.5)	
Diastolic blood pressure				
Hypotension	0 (0.0)	5 (1.3)	0 (0.0)	0.055^a^
Normal	11 (64.7)	248 (62.9)	70 (49.3)	
Hypertension	7 (41.2)	141 (35.8)	71 (50.0)	
Fever	10 (55.6)	289 (72.4)	125 (85.0)	0.002^b^^,^ ^c^
Cough	11 (61.1)	248 (62.2)	76 (51.7)	0.087^c^
Dyspnea	14 (77.8)	313 (78.4)	138 (93.9)	0.0001^b^^,^ ^c^
Sputum production	0 (0.0)	44 (11.0)	10 (6.8)	0.123^a^
Chest pain	4 (22.2)	204 (51.1)	96 (65.3)	0.0001^b^^,^ ^c^
Nausea	5 (27.8)	83 (20.8)	31 (21.1)	0.777^c^
Vomiting	2 (11.1)	74 (18.5)	33 (22.4)	0.396^a^
Diarrhea	5 (27.8)	35 (8.8)	12 (8.2)	0.021^b^^,^ ^c^
Rhinorrhea	0 (0.0)	3 (0.8)	1 (0.7)	0.932^a^
Abdominal pain	0 (0.0)	23 (5.8)	12 (8.2)	0.318^a^
Conjunctivitis	0 (0.0)	1 (0.3)	2 (1.4)	0.272^a^
Headache	5 (27.8)	214 (53.6)	82 (55.8)	0.078^c^
Myalgia	7 (38.9)	148 (37.1)	65 (44.2)	0.318^c^
Arthralgia	2 (11.1)	36 (9.0)	35 (23.8)	0.000^a^^,^ ^b^
Fatigue	6 (33.3)	121 (30.3)	32 (21.8)	0.127^c^
Sore throat	3 (16.8)	21 (5.3)	7 (4.8)	0.104^a^
Chills	4 (22.2)	35 (8.8)	8 (5.4)	0.044^a^
Confusion	0 (0.0)	63 (15.8)	27 (18.4)	0.131^a^
Anosmia	0 (0.0)	19 (4.8)	3 (2.0)	0.237^a^

^a^ The Monte Carlo test was used for the comparison.

^b^ Significant difference.

^c^ The Chi square test was used for the comparison.

A comparison of hematological indices between disease severity groups in the study patients is presented in Table 4. There was a significant difference in lymphopenia (*p* = 0.014), WBC count (*p* = 0.001), neutrophil count (*p* < 0.0001), lymphocyte count (*p* < 0.0001), and prothrombin time (PT) (*p* = 0.001) between disease severity groups.

**Table 4:** Comparison of hematological indices between disease severity groups in patients in the study.

Variable	Mild/moderate	Severe	Critical	
Categorical variables	Frequency (%)	Frequency (%)	Frequency (%)	*p* value
Leucocyte count				
Leukopenia	1 (5.9)	33 (8.3)	14 (9.5)	0.168^a^
Normal	12 (70.6)	228 (57.3)	69 (46.9)	
Leukocytosis	4 (23.5)	127 (34.4)	64 (34.5)	
Neutropenia	0 (0.0)	4 (1.0)	2 (1.4)	0.589^a^
Lymphopenia	10 (62.5)	303 (80.2)	126 (88.1)	0.014^b^^,^ ^c^
Anemia	4 (23.5)	128 (32.2)	51 (34.9)	0.601^a^
Thrombocytopenia	0 (0.0)	32 (8.1)	16 (10.9)	0.255^a^
RDW > 14.5%	4 (22.2)	141 (35.3)	55 (37.4)	0.443^a^
RDW ≤ 14.5%	11 (61.1)	271 (67.9)	95 (64.6)	0.670^b^
**Continuous variables** d	**Median (IQR)**	**Median (IQR)**	**Median (IQR)**	***p***
WBC count (×10^3^)	7.9 (4.0)^e^	8.0 (6.5)^f^	10.6 (7.5)^f^^,^ ^e^	0.001^c^
Neutrophil count (×10^3^)	80.0 (15.0)^g^^,^ ^h^	82.0 (13.1)^g^^,^ ^i^	87.6 (7.7)^g^^,^ ^i^ ^,^ ^h^	<0.0001^c^
Lymphocyte count (×10^3^)	15.0 (12.0)^j^^,^ ^k^	13.0 (11.9)^j^^,^ ^l^	8.5 (6.9)^l^^,^ ^k^	<0.0001^c^
Hb (mg/dL)	12.9 (3.2)	13.1 (3.1)	13.4 (2.2)	0.081
Platelet count (×10^3^)	210.0 (123.0)	190.0 (102.7)	204.5 (137.7)	0.254
RDW (%)	14.0 (2.6)	13.9 (2.4)	14.0 (2.0)	0.550
PDW (fL)	13.3 (2.7)	13.4 (2.7)	13.3 (3.1)	0.434
PT	12.4 (1.6)^m^	12.6 (2.0)^n^	11.6 (2.0)^n^^,^ ^m^	0.001^c^
PTT	30.0 (7.0)	30.0 (5.0)	30.0 (10.2)	0.887
INR	1.0 (0.2)	1.1 (0.2)	1.0 (0.2)	0.253

Abbreviations: Hb, hemoglobin; INR, international normalized ratio; IQR, interquartile range; PDW, platelet distribution width; PT, prothrombin time; PTT, partial thromboplastin time; RDW, red cell distribution width; WBC, white blood cell.

^a^ The Monte Carlo test was used for the comparison.

^b^ The Chi square test was used for the comparison.

^c^ Significant difference.

^d^ The Kruskal–Wallis test was used for the comparison. The Mann–Whitney test was used as post hoc comparison.

^e^ *p* < 0.0001.

^f^ *p* = 0.001.

^g^ *p* = 0.031.

^h^ *p* < 0.0001.

^i^ *p* < 0.0001.

^j^ *p* = 0.036.

^k^ *p* < 0.0001.

^l^ *p* < 0.0001.

^m^ *p* < 0.0001.

^n^ *p* = 0.002.

Binary logistic regression was performed to identify the parameters that could predict severe and critical COVID‐19 (Table 5). The regression analysis revealed that pulse rate (*p* = 0.030, cOR = 1.014, 95% CI for cOR: 1.001, 1.027) and Leukopenia (*p* = 0.016, cOR = 3.910, 95% CI for OR: 1.294, 11.809) could significantly predict severe COVID‐19 infection. This finding indicates that the odds of having severe COVID‐19 increases by 1.014‐fold by one beat/minute increase in pulse rate and the odds of severe COVID‐19 infection increases by 3.190‐fold if patients develop Leukopenia. The regression analysis also revealed that pulse rate (*p* < 0.0001, cOR = 1.075, 95% CI for cOR: 1.046, 1.104), fever (*p* = 0.045, cOR = 2.516, 95% CI for cOR: 1.020, 6.203), dyspnea (*p* = 0.022, cOR = 4.190, 95% CI for cOR: 1.227, 14.306), and leukocytosis (*p* < 0.0001, cOR = 3.866, 95% CI for cOR: 1.815, 8.236) could significantly predict critical COVID‐19 infection. This finding indicates that the odds of having critical COVID‐19 increase by 1.075‐fold by one beat/minute increase in pulse rate and the odds of critical COVID‐19 infection increase by 2.516, 4.190, and 3.866‐fold if patients develop fever, dyspnea, and leukocytosis, respectively. Additionally, the adjusted multivariable regression analysis based on age, gender, diabetes mellitus, and hypertension revealed that Leukocytosis could significantly predict severity of the COVID‐19 in both severe (*p* = 0.022, adjusted odds ratio [aOR] = 0.601, 95% CI for aOR: 0.389–0.928) and critical (*p* = 0.014, aOR = 0.575, 95% CI for aOR: 0.369–0.894) groups. Detailed adjusted analyses based on the patient's age, gender, diabetes mellitus, and hypertension are presented in Table 6.

**Table 5:** Relationship between selected study variables and severe and critical COVID‐19.

Variable	Severe				Critical			
	cOR	95% CI for OR		*p* value	cOR	95% CI for OR		*p* value
		Lower	Upper			Lower	Upper	
Pulse rate	1.014	1.001	1.027	0.030^a^	1.075	1.046	1.104	<0.0001^a^
Systolic BP	1.008	0.988	1.029	0.427	0.993	0.963	1.023	0.634
Diastolic BP	1.006	0.972	1.041	0.751	1.024	0.971	1.080	0.382
Hb	1.008	0.921	1.104	0.861	1.070	0.952	1.203	0.255
PT	0.991	0.949	1.034	0.672	0.987	0.926	1.053	0.692
Fever	1.374	0.822	2.294	0.225	2.516	1.020	6.203	0.045^a^
Cough	1.242	0.757	2.038	0.391	1.570	0.758	3.252	0.225
Dyspnea	1.368	0.747	2.507	0.310	4.190	1.227	14.306	0.022^a^
Sputum production	0.863	0.413	1.801	0.694	0.676	0.186	2.462	0.553
Chest pain	1.451	0.889	2.370	0.137	1.849	0.888	3.852	0.101
Diarrhea	1.340	0.542	3.314	0.526	2.684	0.742	9.702	0.132
Fatigue	0.934	0.552	1.581	0.799	0.723	0.325	1.609	0.427
Sore throat	0.992	0.361	2.723	0.987	0.699	0.109	4.485	0.706
Chills	1.155	0.515	2.589	0.727	1.554	0.406	5.942	0519
Confusion	0.964	0.487	1.911	0.917	0.637	0.236	1.718	0.373
Leukopenia	3.910	1.294	11.809	0.016^a^	3.660	0.770	17.405	0.103
Leukocytosis	1.585	0.929	2.704	0.091	3.866	1.815	8.236	<0.0001^a^

Abbreviations: CI, confidence interval; cOR, crude odds ratio.

a Significant difference.

**Table 6:** Relationship between selected study variables and severe and critical COVID‐19 adjusted based on the age, gender, diabetes mellitus, and hypertension.

Variable	Severe				Critical			
	aOR	95% CI for OR		*p* value	aOR	95% CI for OR		*p* value
		Lower	Upper			Lower	Upper	
Pulse rate	1.038	1.022	1.053	0.000^a^	1.046	1.030	1.062	0.000^a^
Systolic BP	1.004	0.987	1.020	0.671	1.002	0.985	1.019	0.812
Diastolic BP	0.984	0.956	1.013	0.281	0.993	0.964	1.023	0.660
Hb	0.967	0.892	1.049	0.420	0.898	0.818	0.986	0.024^a^
PT	1.000	0.998	1.002	0.963	0.999	0.997	1.001	0.535
Fever	0.561	0.340	0.926	0.024^a^	0.609	0.370	1.001	0.050
Cough	1.019	0.665	1.561	0.931	1.342	0.877	2.052	0.176
Dyspnea	0.434	0.227	0.832	0.012^a^	0.386	0.202	0.738	0.004^a^
Sputum production	1.929	0.830	4.485	0.127	1.841	0.820	4.133	0.139
Chest pain	0.795	0.514	1.230	0.304	0.695	0.450	1.074	0.101
Diarrhea	1.243	0.596	2.594	0.562	0.889	0.431	1.833	0.750
Fatigue	0.936	0.595	1.473	0.776	1.119	0.704	1.778	0.636
Sore throat	0.807	0.302	2.156	0.669	1.430	0.487	4.193	0.515
Chills	0.753	0.334	1.697	0.493	1.460	0.594	3.588	0.410
Confusion	1.239	0.704	2.181	0.457	0.633	0.356	1.125	0.119
Leukopenia	1.248	0.544	2.862	0.601	0.523	0.242	1.128	0.098
Leukocytosis	0.601	0.389	0.928	0.022^a^	0.014^a^	0.575	0.369	0.894

Abbreviations: aOR, adjusted odds ratio; CI, confidence interval.

a Significant difference.

## 4 DISCUSSION

According to the represented results of our study, the findings indicated that the frequency of bradycardia was significantly higher in severe and critical patients compared to mild/moderate patients, while the prevalence of tachycardia significantly increased with the worsening of disease severity. Similarly, the prevalence of fever, dyspnea, arthralgia, lymphopenia, and the mean WBC count, and lymphocyte count increased with the increased severity of the disease, while the frequency of chills, diarrhea, and the mean PT was higher in severe patients compared to critical patients. The risk factors for severe and critical disease were pulse rate, fever, dyspnea, leukopenia, and leukocytosis.

To have a detailed view of the clinical manifestations of COVID‐19, our results revealed that among the study parameters, pulse rate, fever, dyspnea, and leukocytosis were the risk factors for severe COVID‐19. Decreased oxygen saturation was the most common severity criterion; however, dyspnea revealed the most significant effect on the critical situation of COVID‐19 (approximately fourfold effect). Additionally, diarrhea and fever were two other factors related to the critical COVID‐19 but not the severe infection. Despite some previous studies have been indicated similar findings, still, there are some major differences to identify the strongest COVID‐19 severity factor. A study by Nabavi et al.^5^ in the northeast of Iran reported that low O_2_ saturation, nausea/vomiting, and extent of lung CT involvement were independent predictors of severe/critical COVID‐19 (OR = 0.342, 45.93, and 25.48, respectively; *p* < 0.05). On the other hand, a study by Allameh et al.^22^ in Tehran, Iran, and another study by Gharebaghi et al.^23^ in the West side of Iran indicated fever (93.59%) and shortness of breath (62.8%) as the most common clinical manifestations of the severe COVID‐19. Interestingly, Sohrabi et al.^24^ revealed that impaired consciousness could be the most powerful predictor of COVID‐19 severity and mortality (OR = 3.73, 95% CI = 3.508–3.979, *p* < 0.001); however, they reported decreased O_2_ saturation and dyspnea as other significant factors (OR = 2.67 and 1.63, respectively). Further studies in other parts of the world presented some different findings. In a previous review article, the predictors of severe COVID‐19 were preexisting renal failure, oxygen requirement during admission, acute kidney injury, and CRP at the time of admission.^25^ This finding was in contrast to the findings of our study. Moreover, dyspnea could be a presentation of the high requirement for oxygen supplementation which could be known in line with the findings of the mentioned study. The reason for this difference might be due to the high percentage of severe and critical patients in our study. In another study on 2772 elder patients with COVID‐19 in Spain, the predictors of mortality were male gender, O_2_ saturation below 90% at admission, fever, organ failure, and chest X‐ray infiltration. The study also indicated that lymphopenia and increased neutrophils were independent risk factors for death in elderly COVID‐19 patients.^26^ These findings were not in line with our study findings. The reason for the difference might be related to the study population's age and the dependent variable for the analysis (mortality vs severe disease). A study conducted on 665 COVID‐19 patients in Tehran, Iran indicated that leukocytosis was one of the predictors for ICU admission.^27^ Considering ICU admission as a clinical sign of disease severity, this finding was in line with the findings of our study.

It has been demonstrated that simple hematologic indexes can predict disease severity in various diseases including COVID‐19.^28^ The findings of the current study showed that the frequency of lymphopenia increased with disease severity in COVID‐19 patients. In a systematic review on a total of 2282 COVID‐19 patients, lymphopenia was considered as a risk factor for severe COVID‐19 and increased the risk of severe disease by threefold.^29^ Additionally, Ghahramani et al.^30^ reported a significant decrease in lymphocyte, monocyte, eosinophil, hemoglobin, platelet, lymphocyte to CRP ratio, leukocyte to CRP ratio, and an increase in the neutrophil, ESR, CRP, PT, D‐dimer, glucose level, and neutrophil to lymphocyte ratio in the severe group compared with the nonsevere group. No significant changes in WBCs were observed in their study.^30^ The findings of our study showed that WBC and neutrophil count were higher in critical COVID‐19 patients compared to patients with mild/moderate disease. This finding was in line with the findings of a previous study.^31^, ^32^, ^33^, ^34^, ^35^ This finding might be attributed to the cytokine storm in COVID‐19 patients in critical condition.^36^ A similar finding was reported in previous studies.^31^, ^37^, ^38^, ^39^, ^40^ Furthermore, a study by Esfahanian et al in Tehran, Iran, revealed that leukopenia and leukocytosis were not correlated with COVID‐19 severity.^41^ The mechanism for lymphopenia in COVID‐19 is not yet known. The findings of the studies on MERS and SARS‐Cov showed that peripheral T‐cells reduce due to sequestration in various organs immediately after infection.^42^ On the other hand, SARS‐Cov‐2 tends to infect cells through angiotensin‐converting enzyme 2 receptor,^43^ which is highly presented on lymphocytes. Therefore, this mechanism might be the reason for the observed lymphopenia in patients with severe COVID‐19.

Despite the present study revealed valuable findings to manage critically ill COVID‐19 patients, there were several limitations including the limited number of participants, lack of long‐term follow‐up, and limited variables measured; therefore, it is suggested to cover these limitations in future studies.

## 5 CONCLUSIONS

The findings revealed that despite the decreased oxygen saturation being the most frequent presentation in severe and critical COVID‐19 patients, leukopenia and leukocytosis have the strongest correlation with the disease severity. These findings could be a valuable guild for clinicians in COVID‐19 patient management in the inpatient setting. However, these findings could be generalizable, in the outpatient setting. Therefore, it is suggested to conduct further studies to find out the gray areas of the COVID‐19 severity parameters and improve disease management to reduce its morbidities and mortalities.

## AUTHOR CONTRIBUTIONS

*Study concept, design, and supervision*: Mahnaz Mozdourian. *Study concept, design, and supervision*: Fateme Sharafi. *Acquisition of data*: Ali Shamshirian. *Analysis and interpretation of data and statistical analysis*: Lida Jarahi. *Drafting of the manuscript and critical revision for important intellectual content*: Reza Jafarzadeh Esfehani and AmirAli Moodi Ghalibaf. All authors have read and approved the final version of the manuscript had full access to all of the data in this study and takes complete responsibility for the integrity of the data and the accuracy of the data analysis.

## CONFLICT OF INTEREST STATEMENT

The authors declare no conflicts of interest.

## ETHICS STATEMENT

The method was approved in terms of compliance with scientific and ethical standards. All methods were performed in line with the relevant guidelines and regulations. The Ethics Committee of Mashhad University of Medical Sciences, Mashhad, Iran, also approved it. The registered ethical number is IR.MUMS.MEDICAL.REC.1399.485.

## TRANSPARENCY STATEMENT

The lead author Mahnaz Mozdourian affirms that this manuscript is an honest, accurate, and transparent account of the study being reported; that no important aspects of the study have been omitted; and that any discrepancies from the study as planned (and, if relevant, registered) have been explained.

## Data Availability

The datasets created during the current study are not publicly accessible due to the possibility of compromising the privacy of individuals. According to the written approval forms accepted by the Ethics Committee of Mashhad University of Medical Sciences (MUMS), the data were only available to researchers within the project. The data would be available upon request from the corresponding authors (according to the MUMS rules and regulations).
